# Artemisinins in Combating Viral Infections Like SARS-CoV-2, Inflammation and Cancers and Options to Meet Increased Global Demand

**DOI:** 10.3389/fpls.2022.780257

**Published:** 2022-02-07

**Authors:** Karim Farmanpour-Kalalagh, Arman Beyraghdar Kashkooli, Alireza Babaei, Ali Rezaei, Alexander R. van der Krol

**Affiliations:** ^1^Department of Horticultural Science, Faculty of Agriculture, Tarbiat Modares University, Tehran, Iran; ^2^Laboratory of Plant Physiology, Wageningen University and Research, Wageningen, Netherlands

**Keywords:** *Artemisia annua*, artemisinin, COVID-19, malaria, SARS-CoV-2, sesquiterpene lactone

## Abstract

Artemisinin is a natural bioactive sesquiterpene lactone containing an unusual endoperoxide 1, 2, 4-trioxane ring. It is derived from the herbal medicinal plant *Artemisia annua* and is best known for its use in treatment of malaria. However, recent studies also indicate the potential for artemisinin and related compounds, commonly referred to as artemisinins, in combating viral infections, inflammation and certain cancers. Moreover, the different potential modes of action of artemisinins make these compounds also potentially relevant to the challenges the world faces in the COVID-19 pandemic. Initial studies indicate positive effects of artemisinin or *Artemisia* spp. extracts to combat SARS-CoV-2 infection or COVID-19 related symptoms and WHO-supervised clinical studies on the potential of artemisinins to combat COVID-19 are now in progress. However, implementing multiple potential new uses of artemisinins will require effective solutions to boost production, either by enhancing synthesis in *A. annua* itself or through biotechnological engineering in alternative biosynthesis platforms. Because of this renewed interest in artemisinin and its derivatives, here we review its modes of action, its potential application in different diseases including COVID-19, its biosynthesis and future options to boost production.

## Graphical Abstract

Three anti-SARS-CoV-2 potentials of artemisinin and artesunate. Artemisinin (ART) and artesunate (AS) may (1) block interaction of viral spike protein with the human ACE2 receptors, preventing viral endocytosis and activation of the NF-κB signaling pathway, (2) ART and AS may block activation of NF-κB signaling pathway by IKK, or (3) may interfere directly with p50/p65 transcriptional activity in human cells.

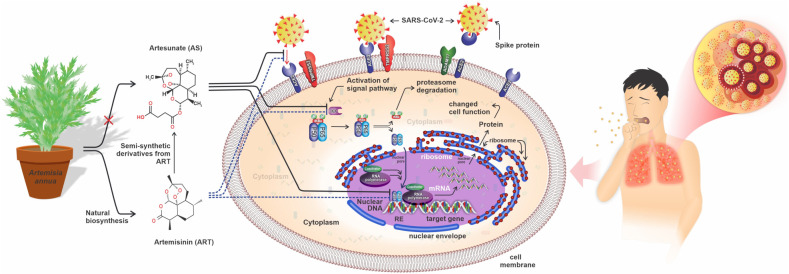

## Introduction

**FIGURE 1:**
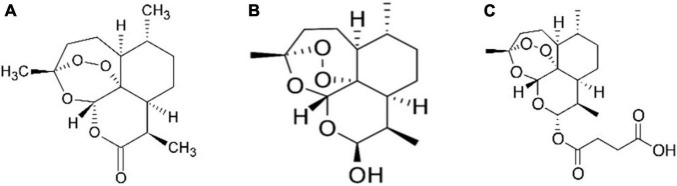
Chemical structure of artemisinin and related compounds: artemisinin **(A)**, dihydroartemisinin, another biosynthetic pathway product and also known as dihydroqinghaosu, or artenimol **(B)** and artesunate, which is a semi-synthetic chemical derivative of artemisinin biosynthetic pathway product **(C)**.

Artemisinin is an oxygenated sesquiterpene lactone, mostly produced in glandular trichomes (GTs) of the medicinal plant *Artemisia annua* L. (Tang et al., 2014; Wang et al., 2016; Beyraghdar Kashkooli et al., 2018, 2019). Artemisinin and related compounds derived from the biosynthetic pathway (Figure 1) have been shown to be effective against malaria caused by the *Plasmodium* spp. parasite (Klayman, 1985; Tu, 2011). The action of artemisinin is not only on the *Plasmodium* itself, but also because of its effect on human physiology. It is the effects of artemisinin on human physiology that relate to its potential uses in other diseases as well. Below the mode of action of artemisinin and related compounds in *Plasmodium* and humans are discussed, exemplified by its potential use in the fight against COVID-19.

Because of the alternative uses of artemisinin, including the fight against COVID-19, the renewed demand for this compound cannot be met by current production capacity. Therefore, also current production capacity problems and potential solutions are briefly discussed.

## Artemisinin: Modes of Action in the Malaria *Plasmodium* Parasitic Cells

**FIGURE 2:**
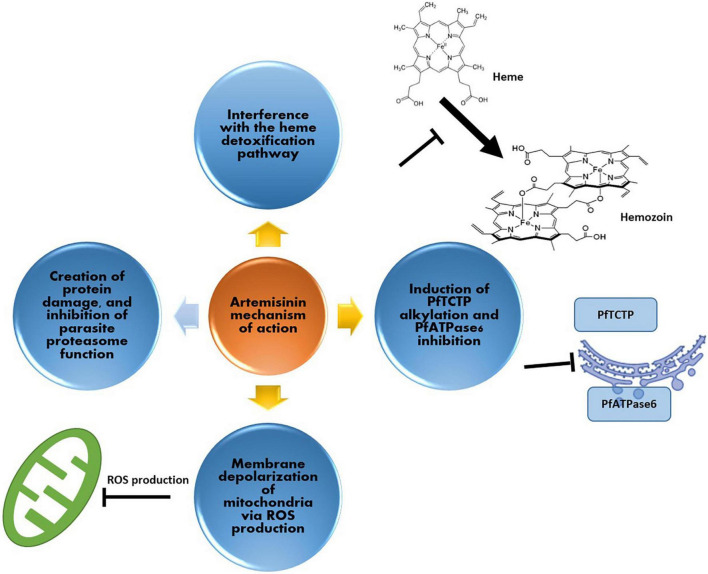
Artemisinin mechanism of action against malaria parasite; (i) production of ROS for depolarization of the parasite’s mitochondria, (ii) interference with the heme detoxification pathway of red blood cells (iii) induction of alkylation and inhibition of cellular elements such as PfATPase6 and (iv) via protein damage, and inhibition of parasite proteasome function.

Artemisinin contains an endoperoxide bridge that is important for anti-malarial activity. In general, several mechanisms of actions have been proposed to explain the bioactivity of artemisinin against *Plasmodium* spp. (O’Neill et al., 2010). The first proposed action is the interference with *Plasmodium* mitochondrial and plasma functions (Antoine et al., 2014). Studies have shown that artemisinin/artemisinin semi-synthetic derivatives (totally known as endoperoxides) induce *Plasmodium* mitochondrial and plasma membrane depolarization (Wang J. et al., 2010; Antoine et al., 2014). These membrane depolarizations are strongly associated with Reactive Oxygen Species (ROS) that are generated by iron bioactivation of the endoperoxides of artemisinin (Antoine et al., 2014; Wang J. et al., 2015; Tilley et al., 2016). An additional mode of action of artemisinin against the onset of malaria is based on the cleavage of the endoperoxide in the artemisinin molecule, resulting in artemisinin free radicals, which act as alkylation agent for susceptible molecules and proteins in the parasitic cell. For instance, the alkylation of *Plasmodium falciparum* TCTP, ATP6 (a Ca^2+^ transporter) (Shandilya et al., 2013) and PI3K (Mbengue et al., 2015) may interfere with the biological function of these proteins in the infection process. In infected red blood cells, the malaria parasite degrades hemoglobin (as a source of amino acids), resulting in large amounts of free heme molecules. These are potentially toxic to the malaria parasite but are detoxified by the parasite via conversion of heme to hemozoin. The alkylation of heme by activated artemisinin could inhibit this detoxification reaction to hemozoin. In a more general sense, the alkylation of parasitic proteins may also interfere with their correct folding, which in turn may be linked to decreased parasite development. Indeed, treatment with artemisinin results in an upregulation of the Unfolded Protein Response (UPR) (Mok et al., 2015). In addition to artemisinin, dihydroartemisinin attacks parasites by using a two-pronged process, creating protein damage, and endangering parasite proteasome function. The consequent gradual accumulation of proteasome substrates (i.e., polyubiquitinated and unfolded/damaged proteins) results in the endoplasmic reticulum stress and dihydroartemisinin-mediated death of the parasite. Tests with other specific inhibitors of the proteasome create a similar increase of polyubiquitinated proteins, also causing parasite death (Bridgford et al., 2018) (Figure 2).

## Artemisinins: Potential Modes of Action in Human Inflammation Responses

Besides its role in combating malaria, artemisinin has also been investigated for its potential effect on immune responses under physiological and pathological conditions (Efferth et al., 2002; Aldieri et al., 2003; Xu et al., 2007; Gu et al., 2012; Lai et al., 2015; Yu et al., 2016; Nunes et al., 2017). Many bacteria and viruses, including the SARS-CoV-2, activate the NF-κB (Nuclear Factor kappa B) signaling pathway in human cells. NF-κB is a transcription factor that regulates multiple aspects of innate and adaptive immune functions and has a central role in inflammatory responses. For instance, NF-κB induces the expression of pro-inflammatory genes like those encoding cytokines and chemokines. NF-κB is a heterodimeric protein complex consisting of p50/p65 which is retained in the cytosol by interaction with IκBα (Verma et al., 1995). Activation of NF-κB signaling activates the IκB kinase activity which results in the release of p50/p65 from IκBα and subsequent movement of p50/p65 to the nucleus where it leads to the expression of specific genes and the production of pro-inflammatory chemokines and cytokines like Interleukin 6 (IL-6) (Verma et al., 1995; Pahl, 1999; Xiong et al., 2010; Liu et al., 2017; Figure 2). IL-6, is a pleiotropic cytokine that is produced in response to infection, tissue-damaging, cellular immune response, and hematopoiesis to contribute and help the host’s defense system (Tanaka et al., 2014; Velazquez-Salinas et al., 2019). Normally, the production of IL-6 is strictly regulated at transcriptional and post-transcriptional levels. However, certain diseases, like in COVID-19, may cause misregulation of the NF-κB signaling, causing overproduction of IL-6 and other cytokines in a cytokine release syndrome (CRS) (Tanaka et al., 2014; Conti and Younes, 2020; Krishna et al., 2021). Indeed, the dynamic change of IL-6 level can be used as a potential biomarker for a severe case of COVID-19 (Liu et al., 2020; Ulhaq and Soraya, 2020; Zhu et al., 2020). In addition to IL-6, also other factors including interferon γ, tumor necrosis factor (TNF), and Interleukin 1 (IL-1), etc., are over-produced during CRS and contribute to pathophysiological processes and multi-organ dysfunction (MOD) (Krishna et al., 2021). The cytokine storm (CS) during CRS may be brought under control by artemisinin or artesunate treatment as these block NF-κB signaling by inhibiting IKK activity (Efferth et al., 2002; Aldieri et al., 2003; Xu et al., 2007; Gu et al., 2012; Lai et al., 2015; Nunes et al., 2017; Efferth and Oesch, 2021). The activation of NF-κB signaling results in the downstream activation of the p50/p65 transcription factors, and artemisinin and artesunate may also act as an inhibitor in the NF-κB signaling pathway by blocking the function of p50/p65 in transcriptional activation of target genes like IL-6.

## Artemisinins: Potential Modes of Action in Fighting Human Cancers

Artemisinins have been used to combat many different types of cancers and different modes of action have been described (Table 1). One of the most important mechanisms is preventing the activation of NF-κB signaling pathway involved in tumor induction, initiation, and progression of many cancerous cell lines. It is noteworthy that artemisinin may affect NF-κB signaling at different levels: it inhibits initiation of nuclear signaling by preventing interaction of p65 and p50 to cytosolic IKK, but in the nucleus it also inhibits interaction of p50 and p65 with target promoters (Tran et al., 2014) (see GRAPHICAL ABSTRACT). Also, *Helicobacter pylori*-induced gastric oncogenesis is inhibited by artemisinins through blocking NF-κB signaling. Remarkably in gastric cancer, artemisinins reverse the IκBα level, prevent NF-κB pathway in a dose-dependent manner, and decrease the generation of downstream inflammatory factors such as TNF-α (tumor necrosis factor-α) and IL-8 (interleukin-8) (Su et al., 2019). The artemisinin related compound dihydroartemisinin induces autophagy via suppressing NF-κB pathway in myeloma, colorectal, and cervical cancer cell lines (Hu et al., 2014), while the anti-invasive activity of dihydroartemisinin may occur through preventing of PKCa/Raf/ERK and JNK phosphorylation and decreasing NF-κB (Hwang et al., 2010). Indeed, increasing IkBα protein and blocking p65 subunit in NF-κB pathway is boosted by dihydroartemisinin (Dong et al., 2014).

**TABLE 1:** Artemisinins effects/mechanisms of action in combating various cancers.

Artemisinins type	Type of cancer	Effects / Mechanism of action	References
Artemisinin	Renal	Inhibition of protein kinase B	Yu et al. (2019)
Artemisinin	Breast	Enhancing of the anti-tumor immune response in 4T1 cancer cells	Cao et al. (2019)
Artemisinin	Breast	Inhibiting osteoclast formation	Li J. et al. (2019)
Artemisinin	Breast	Decreasing functional levels of estrogen receptor-alpha and ablating estrogen-induced proliferation	Sundar et al. (2008)
Artemisinin	Breast	Delaying the development of 7,12-dimethylbenz[a]anthracene (DMBA)	Lai and Singh (2006)
Artemisinin	Breast	Downregulating expression of the E2F1 transcription factor and loss of E2F1-target cell cycle genes	Tin et al. (2012)
Artemisinin	Breast	Reducing the number of regulatory T cells	Langroudi et al. (2010)
Artemisinin+Transferrin	Breast	Retarding growth of cancer tumors	Lai et al. (2009)
Artemisinin	Fibrosarcoma tumors	Inducing apoptosis in cancer cells	Singh and Lai (2004)
Artemisinin	Ovarian	Inducing reversal of EMT	Liang et al. (2019)
Artemisinin	Prostate	Blocks cancer growth and cell cycle progression by disrupting sp1 interactions with the cyclin-dependent kinase-4 (CDK4) promoter and inhibiting CDK4 gene expression	Willoughby et al. (2009)
Artemisinin	Colon	Inducing doxorubicin resistance in cancer cells via calcium-dependent activation of HIF-1α and P-glycoprotein	Riganti et al. (2009)
Artemisinin	Cervical	Repressing telomerase subunits and inducing apoptosis	Mondal and Chatterji (2015)
Artemisinin	Ishikawa endometrial	Triggering a G1 cell cycle arrest of cancer cells, inhibiting cyclin dependent kinase-4 promoter activity and expression by disrupting NF-kB transcriptional signaling	Tran et al. (2014)
Artemisinin	Neuroblastoma	Reducing cell proliferation and inducing apoptosis	Zhu et al. (2014)
Artemisinin	Nasopharyngeal	Down-regulation of BMI-1 cooperates	Wu J. et al. (2011)
Artemisinin	Gastric	Upregulation of p53	Zhang et al. (2014)
Artemisinins	Various Cancers	Inducing iron-dependent cell death (ferroptosis) in tumor cells	Ooko et al. (2015)
Artemisinins	Various Cancers	Inhibition of tumor angiogenesis	Anfosso et al. (2006)
Artemisinins	Colorectal	Stimulating DR5-specific TRAIL-induced apoptosis by regulating wild type P53	Zhou et al. (2020b)
Artemisinins	Gastric	Inhibition of NF-κB signaling	Su et al. (2019)
Artemisinin+Hyperbaric Oxygen	Leukemia	Decreasing growth rate of cancer cells	Ohgami et al. (2010)
6-Aza-artemisinin	Various Cancers	*In vitro* cell-growth inhibitory activities	Koi et al. (2020)
Artemisinin+Estrogen	Breast and Cervical	Antiproliferative activity	Fröhlich et al. (2018)
Artemisinin+Artemisinin dimer	Breast and Prostate	Inducing declines in proteins involved in apoptosis (survivin), cell cycling (cyclin D1), oncogenesis [c-myelocytomatosis oncogene product (c-MYC)], and dysregulated WNT signaling (beta-catenin)	Gong et al. (2013)
Artemisinin-tagged Holotransferrin	Leukemia	Killing cancer cells	Lai et al. (2005)
Artemisinin+Artesunate	Lung	Elevating intracellular ROS and DNA damage	Li et al. (2018)
Artemisinin+Transferrin	Prostate	Induction of apoptosis	Nakase et al. (2009)
Artesunate	Cancer stem cells	Mitochondrial dysfunction of stem cells	Subedi et al. (2016)
Artesunate	Prostate	Targeting NF-kappa B Signaling	Nunes et al. (2017)
Artesunate	Prostate	Suppressing the viability and mobility of cancer cells through UCA1, the sponge of miR-184	Zhou et al. (2017)
Artesunate	Head and Neck	Inducing ferroptosis in cancer cells by decreasing cellular GSH levels, increasing lipid ROS levels, and activation of Nrf2–antioxidant response element pathway in cancer cells	Roh et al. (2017)
Artesunate	HeLa cervical cancer cells	Mitochondrial fission, autophagy induction, and activating of the PINK1-dependent pathway	Zhang et al. (2018)
Artesunate	Cervical	Inhibiting PGE2 production and Foxp3 expression	Zhang et al. (2014)
Artesunate	Cervical	Inducing radiosensitivity	Luo et al. (2014)
Artesunate	Cervical	Enhancing TRAIL-induced apoptosis in cancer cells through inhibition of the NF-κB and PI3K/Akt signaling pathways	Thanaketpaisarn et al. (2011)
Artesunate	Colorectal	Reducing Ki67 and increasing CD31 expression	Krishna et al. (2015)
Artesunate	Colorectal	Down-regulating immunosuppression from Colon26 and RKO cells by decreasing transforming growth factor β1 and interleukin-10	Cui et al. (2015)
Artesunate	Colorectal	Expression of beta-catenin and E-cadherin	Li et al. (2008)
Artesunate	Colorectal	Attenuating the growth of cancer cells and inhibiting hyperactive Wnt/b-catenin pathway	Li et al. (2007)
Artesunate	Colorectal	Suppressing inflammation and oxidative stress	Kumar et al. (2019)
Artesunate	Colorectal	Activating the intrinsic apoptosis of HCT116 cells through the suppression of fatty acid synthesis and the NF-κB Pathway	Chen et al. (2017)
Artesunate	Colorectal	Down-regulating of β-catenin, suppressing of angiogenesis, cellular proliferating and inducing of apoptosis	Verma et al. (2017)
Artesunate	Bladder	Inducing autophagy dependent apoptosis through upregulating ROS and activating AMPK-mTOR-ULK1 axis	(Zhou et al., 2020a)
Artesunate	Leukemia	Inhibiting angiogenesis and down-regulating vascular endothelial growth factor expression	Zhou et al. (2007)
Artesunate	T-cell leukemia/lymphoma	Increasing of intracellular ROS and activation of the DNA damage marker γ-H2AX	Ishikawa et al. (2020)
Artesunate	Skin	Induction of G0/G1 cell cycle arrest and iron-mediated mitochondrial apoptosis	Jiang et al. (2012)
Artesunate	Liver	Inducing G0/G1 cell cycle arrest and apoptosis via increasing intracellular ROS	Yin et al. (2020)
Artesunate	Liver	Mitigating proliferation of tumor cells by alkylating heme-harboring nitric oxide synthase	Zeng and Zhang (2011)
Artesunate	Laryngeal	Reducing of tumor proliferation	Singh and Verma (2002)
Artesunate	Ovarian	Promoting Th1 differentiation from CD4+ T cells to enhance cell apoptosis via miR-142	Chen et al. (2019)
Artesunate	Ovarian	sensitizing cancer cells to cisplatin by downregulating RAD51	Wang B. et al. (2015)
Artesunate	Ovarian	Inhibiting cancer cell growth and proliferation	Greenshields et al. (2017)
Artesunate	Ovarian	Reducing cell viability	McDowell et al. (2021)
Artesunate	Glioblastoma	Inducing oxidative DNA damage, sustaining DNA double-strand breaks, and the ATM/ATR damage response	Berdelle et al. (2011)
Artesunate	Rhabdomyosarcoma	Inducing ROS and p38 MAPK-mediated apoptosis and counteracting tumor growth	Beccafico et al. (2015)
Artesunate	Merkel cell carcinoma	Affecting T antigen expression and repressing growth and survival of MCPyV-positive cancer cells	Sarma et al. (2020)
Artesunate	Breast	Inhibition of the growth of MCF-7 tumor cell	Dong and Wang (2014)
Artesunate	Breast	Inducing apoptosis pathway by loading into lipid carriers	Tran et al. (2016)
Artesunate	Breast	Induction of apoptosis	Jamalzadeh et al. (2017)
Artesunate	Breast	Enhancing the efficacy of 5-ALA-based SDT	Osaki et al. (2017)
Artesunate	Breast	Activating mitochondrial apoptosis in cancer cells via iron-catalyzed lysosomal ROS production	Hamacher-Brady et al. (2011)
Artesunate	Breast	Inducing G2/M cell cycle arrest through autophagy induction	Chen et al. (2014)
Artesunate	Breast	Down-regulating the expression of Bcl-2 and HSP70, Enhancing the expression of cleaved caspase-9 in MCF-7 and 4T1 cells	Pirali et al. (2020)
Artesunate	Breast	Promoting G2/M cell cycle arrest in MCF7 cancer cells through ATM activation	Wen et al. (2018)
Artesunate	Endometrial	Suppressing the proliferation and development of estrogen receptor-α-positive in HAND2-dependent pathway	Yin et al. (2021)
Artesunate	Lung	Inhibiting invasion and *in vivo* metastasis in cancer cells by targeting essential extracellular proteases	Rasheed et al. (2010)
Artesunate	Lung	Expression of EGFR and ABCG2	Ma et al. (2011)
Artesunate	Bladder	Inducing apoptosis of cancer cells by miR-16 regulation of COX-2 expression	Zuo et al. (2014)
Artesunate	Bladder	Impairing growth in cisplatin-resistant cancer cells by cell cycle arrest, apoptosis and autophagy induction	Zhao et al. (2020)
Artesunate	Colon	Enhancing ablation effect on xenograft cancer cells	Hao et al. (2020)
Artesunate	Colon	Inducing apoptosis and autophagy	Jiang et al. (2018)
Artesunate	Nitrosodiethylamine mediated experimental hepatocellular model	Suppression of IL-6-JAK-STAT signaling	Ilamathi et al. (2016)
Artesunate	HeLa and HepG2 cells	Inducing cell death in cancer cells via enhancing lysosomal function and lysosomal degradation of ferritin	Yang et al. (2014)
Artesunate	Non-small-cell lung	Inhibiting epithelial-mesenchymal transition in cancer cells by down-regulating the expression of *BTBD7*	Wang et al. (2020)
Artesunate	Non-small cell lung	Enhancing radiosensitivity cancer cells via increasing NO production to induce cell cycle arrest at G2/M phase	Zhao et al. (2011)
Artesunate	Pancreatic	Inducing AsPC-1 and PaTU8988 cell death	Wang K. et al. (2019)
Artesunate	Pancreatic	Activating of ferroptosis	Eling et al. (2015)
Artesunate	Gastric		Wang et al. (2017)
Artesunate	Gastric	Inhibiting the growth of cancer cells through the mechanism of promoting oncosis	Zhou et al. (2013)
Artesunate	Gastric	Inhibiting cancer cell growth and inducing apoptosis by down-regulating COX-2	Zhang et al. (2015)
Artesunate	B-cell lymphoma	Suppressing cancer cell growth and metabolism	Våtsveen et al. (2018)
Artesunate	Bone metastasis	Suppressing RANKL-induced osteoclastogenesis through inhibition of PLCγ1-Ca^2+^ - NFATc1 signaling pathway and preventing ovariectomy-induced bone loss	Zeng et al. (2017)
Artesunate	Esophageal	Cell apoptosis and suppressing the proliferation	Shi et al. (2015)
Artesunate	Esophageal	Enhancing radiosensitivity of cancer cells by inhibiting the repair of DNA damage	Fei et al. (2018)
Artesunate	Dermal fibroblasts	Inhibiting myofibroblast formation via induction of apoptosis and antagonism of pro-fibrotic gene expression	Larson et al. (2019)
Artesunate+Histone Deacetylase Inhibitors	Hepatocellular, Colorectal, Lung, and Pancreatic	Elevating heme synthesis via synergistic upregulation of ALAS1 expression	Chen et al. (2019)
Artesunate+Ferrous iron	Leukemia and Astrocytoma	Induction of apoptosis	Efferth et al. (2004)
Artesunate+Sorafenib	Liver	Inhibiting cancer cell growth and apoptosis induction	Li H. et al. (2019)
Artesunate+Cisplatin	Lung	Inhibiting MAPK pathway	Li W. et al. (2021)
Artesunate and Dihydroartemisinin	Neuroblastoma	Inducing apoptosis and ROS in cancer cells	Michaelis et al. (2010)
Artesunate+Connexin-43	Renal and Breast	DNA damage and enhancing the bystander apoptosis of the neighboring cells	Raza et al. (2017)
Artesunate+Allicin	Osteosarcoma	Inhibiting cell proliferation and apoptosis	Jiang et al. (2013)
Artesunate and Dihydroartemisinin	Epithelial ovarian	Inhibiting epithelial ovarian cancer cells via autophagy-mediated cell cycle arrest and suppressing the cell cycle-related NF-κB-signaling pathway	Li et al. (2018)
Dihydroartemisinin	Colorectal	Potentiation of 5-fluorouracil antitumor activity	Yao et al. (2018)
Dihydroartemisinin	Colorectal	Induction of iron-dependent endoplasmic reticulum stress	Lu et al. (2011)
Dihydroartemisinin	HeLa cervical cancer cells	Autophagy within cancer cells through Bcl-2 phosphorylation at Ser70	Wang L. et al. (2019)
Dihydroartemisinin	Cervical	Cytotoxic activity against papillomavirus-expressing epithelial cells	Disbrow et al. (2005)
Dihydroartemisinin	Esophageal	Inactivating of NF-κB in Eca109 and Ec9706	Li et al. (2014)
Dihydroartemisinin	Esophageal	Increasing the sensitivity of photodynamic therapy via NF-κB/HIF-1α/VEGF pathway	Li et al. (2018)
Dihydroartemisinin	Breast	Inducing apoptosis	Mao et al. (2013)
Dihydroartemisinin	Hepatocellular	Inhibiting proliferation and inducing apoptosis of cancer cell by upregulating tumor necrosis factor via JNK/NF-κB pathways	Wu et al. (2019)
Dihydroartemisinin	Ovarian	Inducing apoptosis and inhibiting proliferation, migration, and invasion in cancer cells via inhibition of the hedgehog signaling pathway	Liu et al. (2018)
Dihydroartemisinin	Ovarian	Inhibiting PDGFRα-positive cancer cell growth and metastasis through inducing degradation of PDGFRα protein	Li et al. (2017)
Dihydroartemisinin	Ovarian	Inhibiting cancer cell growth, inducing apoptosis and G2 cell cycle arrest, decreasing of Bcl-xL and Bcl-2, and increasing of Bax and Bad	Jiao et al. (2007)
Dihydroartemisinin	Various Cancers	Inhibiting angiogenesis	Chen et al. (2003)
Dihydroartemisinin	Cholangiocarcinoma and Hepatocarcinoma	Expression of *TDR1*, *MDR1*, *MRP1*, *MRP2*, and *MRP3*	Chaijaroenkul et al. (2011)
Dihydroartemisinin	Pancreatic	Inhibiting cell viability, downregulating the expression of proliferating cell nuclear antigen and cyclin D1, upregulated p21WAF1/CIP1, inducing apoptosis by reducing the ratio of Bcl-2/Bax and increasing the activation of caspase-9	Chen et al. (2009)
Dihydroartemisinin	Pancreatic	Inducing oncosis-like cell death	Du et al. (2010)
Dihydroartemisinin	Pancreatic	Inducing cell cycle arrest, apoptosis, and inhibiting of NF-kB signaling	Chen et al. (2010)
Dihydroartemisinin	Pancreatic	Inhibiting NF-kB pathway	Wang et al. (2011); Wang et al. (2010)
Dihydroartemisinin	Non-small-cell lung	Suppressing metastasis of cancer via inhibiting NF-κB/GLUT1 axis	Jiang J. et al. (2016)
Artemisone	Melanoma	Inhibiting cancer cell growth	Dwivedi et al. (2015)
Artemisone	Breast, Colon, Melanoma, and Pancreatic	Reducing cell viability and arresting cell cycling	Gravett et al. (2011)
Artemether	Gastric	Increasing of DNA-damage index, inducing necrosis in PG100, inducing both apoptosis and necrosis in lymphocytes	Alcântara et al. (2013)
Artesunic acid+Thymoquinone	Colorectal	Increasing of ROS, and elevating levels of DNA-damage marker γ-H2AX	Fröhlich et al. (2017)
Anhydro dihydroartemisinin and 10-dihydroartemisinyl acetate	Liver/Colon	Antiproliferative and inhibiting the release of BVDV-RNA	Blazquez et al. (2013)
Artemisinin, Dihydroartemisinin, and Artesunate	Non-small-cell lung	Inhibiting tumorigenesis and tumor metastasis through Wnt/β-catenin signaling	Tong et al. (2016)

Artesunate attenuates the growth of cancer cells and thus the development of the tumor by targeting NF-κB pathway (Table 1). In prostate cancer cells, resistance to androgen receptor antagonists is reduced by using artesunate. Mechanistically, the combination of artesunate and bicalutamide prevents NF-κB pathway by ubiquitin-mediated proteasomal deterioration (Nunes et al., 2017). In attempts to treat cervical cancer, the evidence demonstrated that artesunate successfully increases tumor necrosis factor-related apoptosis-inducing ligand (TRAIL)-mediated cytotoxicity via pro-survival proteins including X-linked inhibitor of apoptosis protein (XIAP), survivin, and B-cell lymphoma-extra-large (Bcl-xL), and reduces the number of survival proteins in HeLa cells. The downregulation of mentioned proteins can be regulated by repressing activation of serine/threonine-protein kinase and NF-κB signaling. Artesunate further prevents TRAIL-influenced transcriptional activity of NF-κB (Thanaketpaisarn et al., 2011).

## Artemisinins: Potentials in Combating COVID-19 and Other Human Viral Infections

### Physiological Effects of Artemisinins or *Artemisia* spp. Plant Extracts

Currently, we are facing the serious challenge of the COVID-19 pandemic, which has disrupted global health and the economy. COVID-19 is a virus-related disease similar to Severe Acute Respiratory Syndrome CoronaVirus (SARS-CoV) (Lu et al., 2020) and is caused by the SARS-CoV-2 (Lai et al., 2020; Singhal, 2020). Unlike with SARS-CoV, patients infected with SARS-CoV-2 initially have mild symptoms and continue their daily activities, but in the meantime are infectious to others (Heymann and Shindo, 2020; Zheng et al., 2020). In some patients, disease symptoms may suddenly increase dramatically due to the development of a CS entitled CRS with hallmarks in the body of inflammation and immunosuppression (Mehta et al., 2020). CRS in COVID-19 patients may result in respiratory failures that create Acute Respiratory Distress Syndrome (ARDS) and MOD (Krishna et al., 2021). While worldwide efforts are aimed at vaccines that may prevent infection by COVID-19, additional medicines that can alleviate the severe symptoms of COVID-19 are still a high priority, also because new viral variants may escape vaccine recognition.

Previously, *in vitro* studies have indicated that the alkylating activity of activated artemisinin (as discussed above in the context of malaria) or the specific structure of artemisinin may have potential in preventing infections by members of the *Herpesviridae* family (e.g., herpes simplex virus type 1, Epstein-Barr virus, human cytomegalovirus), hepatitis B virus, hepatitis C virus, and bovine viral diarrhea virus (Efferth et al., 2008; Efferth, 2018). Artemisinin also has received renewed attention to fight emerging new viruses for which no effective antiviral drugs are available (e.g., HIV, dengue virus, chikungunya virus, Ebola virus), against viral strains that have developed drug resistance (e.g., human cytomegalovirus) and most recently against Corona-virus (D’alessandro et al., 2020). To combat infection by COVID-19, extracts from different medicinal plants (including from *Artemisia* spp.) have been tested against COVID-19 (Bahrami et al., 2020; Bailly and Vergoten, 2020; Huang et al., 2020; Kapepula et al., 2020; Koshak and Koshak, 2020; Mani et al., 2020; Verma et al., 2020; Williamson and Kerimi, 2020; Belhassan et al., 2021; Hassanipour et al., 2021; Javed et al., 2021; Lyu et al., 2021; Nazrul Islam et al., 2021; Song et al., 2021).

*In vitro* efficacy of artemisinin-based treatments to combating SARS-CoV-2 has indicated that treatment with artesunate, artemether, *A. annua* extracts, and artemisinin hindered virus infections of human lung cancer A549-hACE2 cells, VeroE6 cells, and human hepatoma Huh7.5 cells. Among these four treatments, artesunate showed the strongest anti-SARS-CoV-2 activity (7–12 μg/mL), followed by artemether (53–98 μg/mL), *A. annua* extracts (83–260 μg/mL), and artemisinin (151 to at least 208 μg/mL). Collectively, time-of-addition experiments in A549-hACE2 cells displayed that artesunate attacked the virus at the post-entry level (Zhou et al., 2021). In parallel with the previous study, dried-leaf hot-water extracts of *A. annua* cultivars including SAM, BUR, A3, and MED revealed *in vitro* anti-SARS-CoV-2 activity against Alpha, Beta, Gamma, Delta, and Kappa variants of the virus. All cultivars in addition to being potent in combating with original wild type WA1 also showed effective potential against mentioned variants. IC90 and IC50 according to measured artemisinin content ranged from 1.4–25.0 μM and 0.3 to 8.4 μM, respectively. Also, the IC90 and IC50 according to dried-leaf weight ranged from 59.5–160.6 μg DW and 11.0 to 67.7 μg DW, respectively (Nair et al., 2022). Alternatively, *Artemisia* spp. Extracts and COVID-Organics drink produced in Madagascar hindered *in vitro* SARS-CoV-2 and Feline coronavirus (FcoV) infections at concentrations that did not influence cell efficacy and viability (Nie et al., 2021).

In another successful *in vitro* study, six monomer compounds including artesunate, artemether, arteannuin B, andrographolide, licochalcone B, and echinatin exerted high anti-SARS-CoV-2 and anti-GX_P2V (pangolin coronavirus) activity (Hu et al., 2021). In addition to the mentioned monomers, it is noteworthy that the quinoline like artemisinins has shown strong anti-SARS-CoV-2 activity (Firestone et al., 2021). Also, the anti-SARS-CoV-2 activity of nine artemisinin-based compounds experimented *in vitro*. Results highlighted that arteannuin B, artesunate, and dihydroartemisinin are the most potent agents in inhibiting virus activity. Also, several artemisinins decreased the generating of the virus nucleocapsid (N) proteins in a dose-dependent manner. It can be concluded that targeting N proteins can be considered as one of the possible options to control viral infection. On the other hand, both lumefantrine and arteannuin B suppressed viral infection following SARS-CoV-2 entry toward the host cells (Cao et al., 2020). In addition to artemisinins monotherapy, *in vitro* inhibition of SARS-CoV-2 replication by artemisinin-based combination therapies (ACTs) in African experimental society indicated that the artesunate-mefloquine exerted high anti-SARS-CoV-2 activity with % inhibition of 72.1 ± 18.3%. Also, other ACTs including artesunate-pyronaridine, artesunate-amodiaquine, dihydroartemisinin-piperaquine, and artemether-lumefantrine displayed the same range of inhibition (27.1 to 34.1 %) (Gendrot et al., 2020). Along with *in vitro* studies of ACTs, molecular docking studies have also demonstrated the anti-SARS-CoV-2 activity of artemisinin-thymoquinone hybrids against the main protease of the virus (de Oliveira et al., 2021).

### Molecular Modes of Action of Artemisinins in Combating COVID-19

#### Blocking Receptor Binding of Spike Protein to Host Cell Surface

While extracts from medicinal plants may show some preliminary efficacy in small scale clinical trials (Dong et al., 2020; reviewed in Orege et al., 2021), these do not clarify where the activity is coming from. For instance, the antiviral and immunomodulation effects of *Artemisia* spp. Extracts, as recently been reviewed (Kshirsagar and Rao, 2021), may not only be due to artemisinins, but also from other potential bioactive compounds like flavonoids, mono- and sesqui-terpenes or tannins in these extracts (Kshirsagar and Rao, 2021). To address efficiency and potential harmful side effects of plant extracts, a more detailed knowledge on the molecular mode of action of individual bioactive molecules is needed (Cheong et al., 2020; Guastalegname and Vallone, 2020). Studies indicate that artesunate, dihydroartemisinin, and artemisinin may act at the cell surface by inhibition of the binding of the SARS-CoV-2 spike protein to cell surface receptors, thus potentially preventing both endocytosis of the virus and activation of the NF-κB signaling pathway (Gendrot et al., 2020; Rolta et al., 2020; Sehailia and Chemat, 2020; Uckun et al., 2021).

However, molecular docking studies indicate that artemisinins may also bind to coronavirus-host proteins such as E protein, helicase protein, N protein, 3CL^PRO^, S protein, nonstructural protein 3 (nsp3), nsp10, nsp14, nsp15, cathepsin-L, and glucose-regulated protein 78 receptor (Fuzimoto, 2021; Ribaudo et al., 2021) and part of the biological activity of artemisinin against COVID-19 may thus also be partially based on inhibiting the function of these viral proteins.

#### Preventing Cytokine Storm by Inhibiting IKK

In addition, artemisinin/and or artesunate may limit CS by inhibiting IKK and thus over-active NF-κB signaling, or it may inhibit the transcriptional activity of p50/p65, released by NF-κB signaling (see GRAPHICAL ABSTRACT). While preliminary studies with artemisinins look promising, researchers have warned that the potential of artemisinins in combating COVID-19 requires further clinical research (Uzun and Toptas, 2020; Krishna et al., 2021).

### Artemisinins-Related Clinical Trials in Combating COVID-19

A total of 16 trials with *Artemisia* spp. Extract, artemisinins, and ACTs have been registered in the US National Library of Medicine^1^ with clinical trial IDs: NCT04530617, NCT04306497, NCT04701606, NCT04695197, NCT04475107, NCT04532931, NCT04374019, NCT05084911, NCT04502342, NCT04801017, NCT04374084, NCT05004753, NCT04387240, NCT04553705, NCT04802382, NCT04382040, and 3 trials in Chinese Clinical Trial Registry (ChiCTR) database^2^ with IDs: ChiCTR2000033049, ChiCTR2000032915, and ChiCTR2000030082 (suspended by the investigator) to combat SARS-CoV-2 infection.

Until December 2021, only one trial registered in https://clinicaltrials.gov/ has been terminated with ID number: NCT04530617. Preliminary results from this study indicate that the agents such as *A. annua* and Camostat mesilate may help reduce the number of hospitalized patients and that the use of artemisinin-piperaquine for treatment of COVID-19 is safe (Li et al., 2020). Therefore, the World Health Organization (WHO) has initiated clinical trials on three promising candidate drugs, including artesunate, to evaluate the anti-inflammatory activity against SARS-CoV-2 (Solidarity Trial PLUS is registered at: ISRCTN83971151). As new mutations occur in the SARS-CoV-2, resulting in new variants including Alpha, Beta, Gamma, Delta, Kappa, and Omicron, combating potential downstream effects of COVID-19 infections remains an important aspect of dealing with the ongoing pandemic, especially when this virus has become endemic.

## Natural Artemisinin Production: Low Yield and Alternatives to Boost Production

**FIGURE 3:**
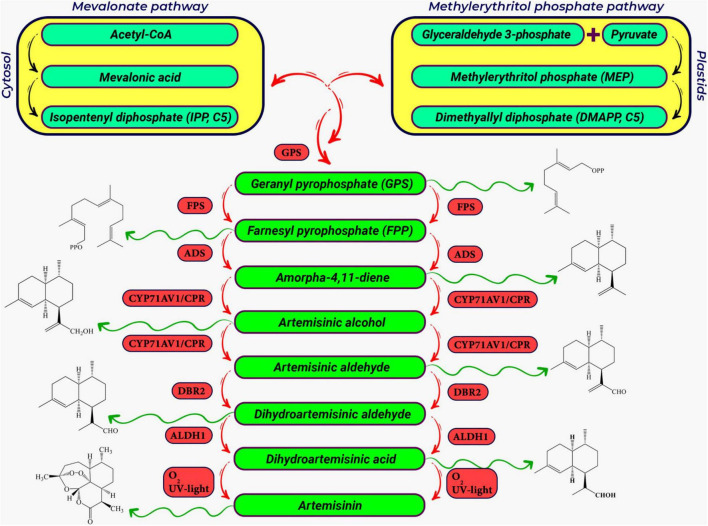
A schematic representation of the artemisinin biosynthetic pathway in *Artemisia annua* L. The precursors for artemisinin biosynthesis (DMAPP and IPP) are produced in the cytosolic Mevalonate (MVA) pathway and the plastidal 2-C-Methylerythritol 4-phosphate pathway (MEP) pathway, respectively (Dewick, 2009; Vranová et al., 2013; Beyraghdar Kashkooli et al., 2019). IPP+DMAPP are converted to FPP, which is the general precursor for sesquiterpenes (Fuentes et al., 2016). In sequential enzymatic steps, FPP is converted to amorphadiene, artemisinin alcohol, artemisinic aldehyde, dihydroartemisinic aldehyde, and finally artemisinic acid. The first and key step in the biosynthesis of artemisinin biosynthetic pathway is the conversion of FPP to amorpha 4, 11-diene (known as amorphadiene), which is catalyzed by a well-known terpene cyclase, the amorpha-4,11-diene synthase (ADS) (Mercke et al., 2000; Bertea et al., 2005). The cytochrome P450 hydroxylase (CYP71AV1) (Teoh et al., 2006) then converts amorphadiene to artemisinic alcohol. CYP71AV1 also oxidizes artemisinic alcohol to artemisinic aldehyde and artemisinic acid, respectively. Artemisinic aldehyde double bond reductase (DBR2) as the branching point and the aldehyde dehydrogenase 1 (ALDH1) (Zhang et al., 2008) convert artemisinic aldehyde to dihydroartemisinic aldehyde and dihydroartemisinic acid, respectively (Bertea et al., 2005; Schramek et al., 2010). Abbreviation for genes in artemisinin biosynthetic pathway includes; GPPS, geranyl pyrophosphate synthase; FPPS, farnesyl pyrophosphate synthase; ADS, armorpha-4, 11-diene synthase; CYP71AV1, cytochrome P450 monooxygenase; CPR, cytochrome P450 reductase; DBR2, artemisinic aldehyde delta-11(13)-double bond reductase; ALDH1, aldehyde dehydrogenase 1.

Artemisinin is produced in glandular trichomes in the leaves and ovary of the *A. annua* (Wang et al., 2016). Both the specificity of artemisinin biosynthesis occurring in GTs and the fact that GTs represent about 2% of plant total weight put a limit to the bulk production of artemisinin *in planta* (Judd et al., 2019). The different steps in artemisinin production are shown in Figure 3. The end-product of the enzymatic pathway (dihydroartemisinic acid) is presumably toxic to the plant cell and is therefore exported over the plasma membrane and the cell wall to a subcuticular space where it is converted non-enzymatically by light (UV) to artemisinin (Wang et al., 2016). In parallel, artemisinic acid, another end-product of the enzymatic pathway may also be exported from the cell, and extracellularly converted to arteannuin B. Typical yield of artemisinin from the *Artemisia annua* plant is 0.6 to 1.2% but may go up to 2% based on plant dry weight (Zhang et al., 2008). However, such yield is low and far from the potential world’s demand (Judd et al., 2019). Current supplies of artemisinin are already limiting to treat people for malaria in a cost-effective way, so use in treating the disease of pandemic proportions like COVID-19 will need new approaches to artemisinin production. Several methods have been proposed to increase artemisinin production so far which are treatments impacting *A. annua* cultivation and physiology and breeding approaches to increase artemisinin yield, engineering artemisinin biosynthetic and transport pathway in the native *A. annua* plants, and engineering of heterologous (plant and microorganism) systems via ectopic expression of the biosynthetic pathway (Lei et al., 2011; Liu et al., 2011; Parshikov et al., 2012; Fuentes et al., 2016; Kiani et al., 2016; Ikram and Simonsen, 2017; Carqueijeiro et al., 2020).

## Treatments Impacting Artemisinin Content in *Artemisia annua*

**FIGURE 4:**
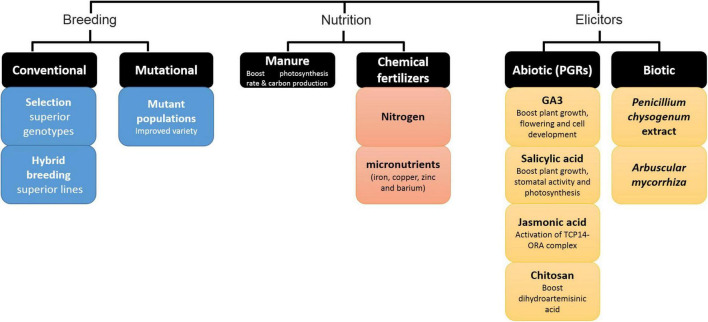
Classical methods to improve artemisinin production in *Artemisia annua.*

Thanks to targeted breeding programs different cultivars of *A. annua* can be grown in a wide range of climate conditions (temperate, cold temperate, subtropical, and Mediterranean) (Ferreira et al., 2005). However, the content and composition of secondary metabolites in *A. annua* plants are determined by numerous interacting factors: geographical conditions, harvesting time, agricultural practices (e.g., fertilization, irrigation, density per unit area) and post-harvest conditions (Mert et al., 2002; Jelodar et al., 2014). Conventional manipulations of the *A. annua* plant that may enhance artemisinin production are discussed below and summarized in Figure 4.

### Nutrient Manipulations

In addition to genetic factors, the artemisinin yield can be influenced by environmental conditions and field management practices (Charles et al., 1991). Furthermore, the use of fertilizer compounds can also affect the artemisinin content. As studies have shown, manure and chemical fertilizers are effective in the production of secondary metabolites by improving the photosynthetic rate and carbon production (Jha et al., 2011). Numerous reports have considered the use of nitrogen (N) fertilizer to be somewhat effective in increasing the artemisinin content (Ferreira et al., 2005; Davies et al., 2009; Aftab et al., 2011). In addition to the important role of macronutrients in increasing the artemisinin production in *Artemisia* plants, studies showed that the deficiency of micronutrients (iron, copper, zinc, and barium) also plays a significant role in reducing the artemisinin content (Srivastava and Sharma, 1990).

### Biotic Elicitors

Similarly, the use of elicitors in plants increases the accumulation of secondary metabolites (Zhao et al., 2005). For instance, the elicitor of *Penicillium chrysogenum* extract has increased (up to double) the production of artemisinin in hairy roots of *A. annua* (Liu et al., 1999). Additionally, the application of chitosan or of the arbuscular mycorrhizal species *Rhizophagus intraradices* (as elicitor) was able to increase the content of dihydroartemisinic acid and artemisinin in *Artemisia* plants (Lei et al., 2011; Mandal et al., 2014). Since the response to these biotic factors often involves the phytohormone jasmonic acid (JA), the effect of these factors on artemisinin content could be due to activated JA signaling.

### Plant Breeding

The main breeding goals of *A. annua* are the improvement of artemisinin production by increasing the yield potential of leaves, proliferate the number of shoots, and raising the total number of glandular trichomes per plant (Graham et al., 2010; Jelodar et al., 2014). Selection through germplasm and genetic modification can be considered as basic strategies for the improvement of artemisinin production in *A. annua* (Charles et al., 1991; Xie et al., 2016). A very common breeding technique to increase the secondary metabolites is the manipulation of the ploidy levels in plants (Weathers, 2003). The production of artificial polyploids as a plant breeding strategy has made it possible to develop new and improved cultivars (Iannicelli et al., 2020). In this regard, the application of ploidy manipulation techniques has successfully increased the artemisinin production in *A. annua*. Reports indicate that the amount of artemisinin in tetraploid plants has increased up to 56% compared to diploid plants. Alternatively, induced mutation using chemicals such as sodium azide (NaN_3_) and ethyl methane sulfonate (EMS) was effective in increasing the artemisinin biosynthesis in native plant (Al-Qurainy and Khan, 2010; Leow et al., 2020).

### Enhancing Glandular Trichomes

In some plant species, the production of some glandular type trichomes is enhanced by JA treatment (Chen et al., 2018) or UVB light (Yan et al., 2012). Indeed, the artemisinin content of *A. annua* is enhanced under the UV treatment at a dosage of 150 gray irradiation (Raymond et al., 2015) and UV-B radiation at 1.44 kJ m^–2^ d^–1^ (Pandey and Pandey-Rai, 2014) respectively. This could be due to both an effect on glandular trichome density and enhanced conversion of artemisinic aldehyde to artemisinin.

### Plant Growth Regulators

Various agricultural practices use plant growth regulators (PGRs) to improve artemisinin production. For example, the treatment of *A. annua* with Salicylic acid increases plant growth, leading to higher biomass (Aftab et al., 2010), altered plant morphology, artemisinin content and composition (Ma et al., 2009). Other studies have shown that PGR GA_3_ (Weathers et al., 2005; Zhang et al., 2005; Aftab et al., 2011) and JA can also increase the artemisinin content of *A. annua* (Zhou and Memelink, 2016). JA has been shown to boost artemisinin biosynthesis via the releasing of repressors of transcription factor TCP14-ORA at the promoters of *double bond reductase 2* (*DBR*) and *aldehyde dehydrogenase 1* (*ADH1*), two key genes in the artemisinin biosynthetic pathway (see Figure 3; Ma et al., 2018).

## Bioengineering of Artemisinin Production in *Artemisia annua*

**FIGURE 5:**
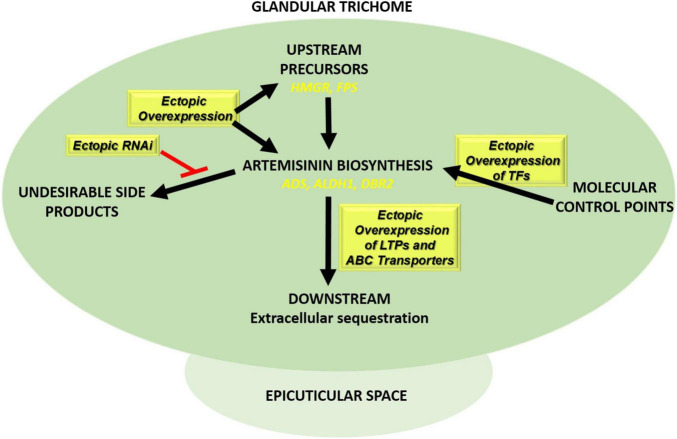
Transformation approaches in *Artemisia annua* plants to boost artemisinin production. Ectopic overexpression of biosynthetic pathway genes (*ADS*, *ALDH1*, and *DBR2*), transcription factors (e.g., AP2/ERF, MYB, WRKY, and bHLH), and genes involved in extracellular sequestration of artemisinin (LTPs/PDRs) together with ectopic RNAi expression of competing pathways such squalene biosynthesis (SQS RNAi).

Metabolic engineering may be used to improve the production of artemisinin in *A. annua* itself but is hampered by the difficulties in efficient transformation and regeneration of *A. annua* plants. Alternatively, the genes that have been isolated from *A. annua* that are involved in artemisinin production may be expressed in a heterologous host that is easier to transform and grow (Covello, 2008; Ma et al., 2015; Xie et al., 2016; Lv et al., 2017; Ikram et al., 2019). Options to manipulate artemisinin production in *A. annua* are briefly discussed below and summarized in Figure 5. The yield effects of the different transformation efforts of *A. annua* are summarized in Table 2.

(1) *Artemisia annua* has been transformed with *Agrobacterium* genes that affect endogenous plant hormone levels (*rol ABC* or *ipt*), resulting in mild to up to 9 times higher artemisinin levels compared to untransformed (or empty vector transformed) control plants (Sa et al., 2001; Bulgakov, 2008; Dilshad et al., 2015; Kiani et al., 2016).
(2) Other transformation strategies are aimed at boosting precursors, either by boosting flux through the Mevalonate pathway by ectopic expression of *3-hydroxy-3-methylglutaryl-CoA reductase* (*HMGR*) or by blocking unwanted side reactions that drain from the precursor pool (e.g., *Squalene synthase* (*SQS*), that diverts FPP to squalene) (Liao et al., 2016). Ectopic expression of *HMGR* in *A. annua* can boost artemisinin production (Aquil et al., 2009; Nafis et al., 2011; Ma et al., 2017), while also suppression of *SQS* can increase artemisinin levels (Paradise et al., 2008; Yang et al., 2008; Zhang et al., 2009; Table 2 and Figure 5).
(3) Transformation approaches may also be aimed at boosting flux through the artemisinin biosynthetic pathway itself through overexpression of biosynthesis genes. Ectopic overexpression of *farnesyl pyrophosphate synthase* (*FPS*) alone or FPS with *CYP71AV1* and *CPR*, increased artemisinin levels in transgenic plants (Table 2; Chen et al., 2000, 2013; Han et al., 2016; Banyai et al., 2010; Wani et al., 2021). In another study, upregulated expression of *HMGR*, *FPS*, *ADS*, *Aldh1*, and *ADS* in *A. annua* increased artemisinin level 39-56% fold (Lin et al., 2011; Figure 5).
(4) The expression of endogenous biosynthesis genes may also be boosted by ectopic overexpression of relevant transcription factors (TF), provided expression of such TF is limiting for transcription of target genes. Multiple TFs (AP2/ERFs, WRKYs, bHLH, MYCs) have been identified in the regulation of endogenous artemisinin biosynthesis genes (Verpoorte and Memelink, 2002; Yang et al., 2012; Shen et al., 2016; Lv et al., 2017), but not all of these have been tested for stable transformation of *A. annua*. However, ectopic expression of WRKY does result in higher artemisinin production in *A. annua* (Jiang W. et al., 2016; Table 2 and Figure 5).
(5) The capacity for extracellular accumulation of dihydroartemisinic acid for extra-cellular conversion to dihydroartemisinin may be of importance for the flux through the biosynthetic pathway to prevent feedback inhibition and possible toxic effects of pathway products. Studies in tobacco have shown that ABC-transporter AaPDR2, in concert with specific LTP AaTLP3 may be required for this function (Wang et al., 2016). In the tobacco assay, *AaLTP3* and *AaPDR2* prevent dihydroartemisinic acid reflux from the apoplast to the cell, resulting in higher artemisinin levels (Wang et al., 2016). However, manipulation of either ABC-transporter or LTP levels in *A. annua* has not been performed till now. Potentially, overexpression of these proteins could result in enhanced artemisinin production in *A. annua* (Figure 5).

**TABLE 2:** Introducing artemisinin (ART) pathway genes in *Artemisia annua* L. to improve the ART production using different strategies.

	Expression type	Yield	References
*Artemisia annua* L.	Overexpression of *HMGR* and *ADS*	1.73 mg/g DW	Alam and Abdin (2011)
*Artemisia annua* L.	Overexpression of *CYP71AV1* and *CPR*	0.98 ± 0.18 mg/g	Shen et al. (2012)
*Artemisia annua* L.	Overexpression of *FPS*,*CYP71AV1* and *CPR*	2.9 mg/g FW	Chen et al. (2013)
*Artemisia annua* L.	Overexpression of *FPS*	1.3% DW	Banyai et al. (2010)
*Artemisia annua* L.	Overexpression of *HMGR*	0.386 ± 0.0332mg/g DW	Aquil et al. (2009)
*Artemisia annua* L.	Suppressing the expression of *SQS*	31.4 mg/g DW	Zhang et al. (2009)
*Artemisia annua* L.	Overexpression of *AaWRKY1*	≥ 14 4 mg/g DW	Jiang W. et al. (2016)
*Artemisia annua* L.	Overexpression of *DBR2*	1.5–2.14 mg/g DW	Yuan et al. (2015)

## Bioengineering of Artemisinin in Heterologous Production Platforms

### *In planta* Artemisinin Production

**FIGURE 6:**
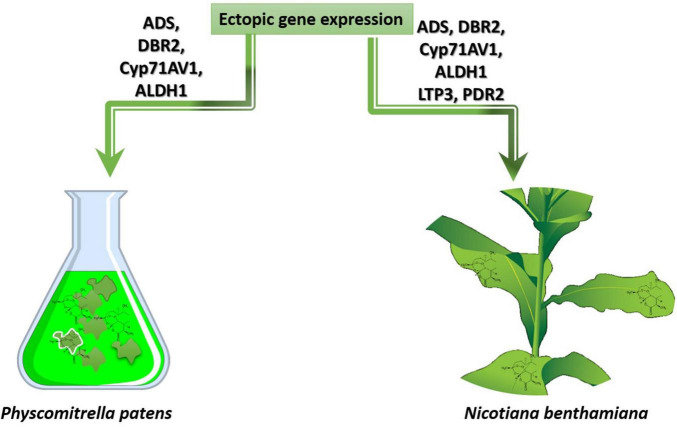
Heterologous overexpression of genes from artemisinin biosynthetic pathway in the host plants *N. benthamiana* and *P. patens*.

The genes for artemisinin production have also been expressed in other plants, either by transient expression or by stable transformation. To date, *in planta* artemisinin production has been reported for tobacco (*Nicotiana benthamiana*) and moss (*Physcomitrella patens*) (Table 3). Transient expression of genes in *N. benthamiana* leaves is used to characterize gene function and has the advantage that up to 15 genes may be co-expressed at the same time to transiently reconstitute entire biosynthetic pathways (Reed et al., 2017; Carqueijeiro et al., 2020). Reconstruction of the artemisinin biosynthetic pathway by transient co-expression of pathway genes (Wallaart et al., 2001; Zhang et al., 2011; Ting et al., 2013; Wang et al., 2016) or stable transformation with pathway genes (Farhi et al., 2011) resulted in the first ectopic production of artemisinin in another plant species (Table 3). Recently, the stable transformation of the moss (*P. patens*) with artemisinin pathway genes, demonstrated that this compound may also be produced in much more primitive plant species (Ikram et al., 2017; Table 3 and Figure 6). As a plant-based production platform of artemisinin, moss has been shown to have promiscuous substrate recognition which may be a substitute for some artemisinin biosynthetic pathway genes which are not present in for example *N. benthamiana*. Substrate promiscuity of sesquiterpenoids pathway from *A. annua* and *Tanacetum parthenium* for individual enzymes or pathways is previously reported (Beyraghdar Kashkooli et al., 2019). Besides, the simple purification step (due to lack of conjugation phenomenon) has been also stated as one of the advantages of this platform compared to the *N. benthamiana*.

**TABLE 3:** Introducing artemisinin (ART) pathway genes in planta to improve the ART production using different strategies.

	Expression type	Yield	References
*Nicotiana benthamiana*	Transient expression of ART precursors’ genes	0.000220347 mg/g FW	Ting et al. (2013)
*Nicotiana benthamiana*	Expression of *ADS*	2e-7-1.7e-6mg/g FW	Wallaart et al. (2001)
*Nicotiana benthamiana*	Stable transformation of *ADS*	0.00048-0.00094 mg ART/g DW	Farhi et al. (2011)
*Nicotiana benthamiana*	Stable transformation of *mtADS*	0.005-0.0068 mg ART/g DW	Farhi et al. (2011)
*Nicotiana benthamiana*	Stable transformation of *ADS*, *CYP71AV1*, and *DBR2*	AD: > 0.004 mg/g FW; AA: > 0.0005 mg/g FW; DA: > 0.0015 mg/g FW	Zhang et al. (2011)
*Nicotiana benthamiana*	Transient expression of *AaLTP3* and *AaPDR2*	0.003 mg/g DW	Wang et al. (2016)
*Nicotiana benthamiana*	SPG Transformation	> 0.12 mg artemisinic acid/g biomass	Fuentes et al. (2016)
*Nicotiana benthamiana*	Stable transformation of ART B.P. genes	0.3–0.8 mg/g DW	Malhotra et al. (2016)
*Nicotiana benthamiana*	Transient expression of ART B.P. genes	0.0395 mg/g FW	van Herpen et al. (2010)
*Physcomitrella patens*	Stable transformation of ART B.P. genes	0.21 mg/g DW	Ikram et al. (2017)

*SPG, Stable Plastid Genome; FW, Fresh Weight; DW, Dry Weight; AD, Amorphadiene; AA, Artemisinic alcohol; DA, Dihydroartemisinic alcohol; ART B.P., Artemisinin biosynthetic pathway. All units are converted to milligram per gram (mg/g).*

### Artemisinin Production in Yeast

Synthetic biology techniques play an important role in the exploration, overproduction, and structure diversification of phytochemicals (Qi et al., 2015; Muhammad et al., 2020; Alam et al., 2021). The full set of artemisinin biosynthesis genes have also been introduced into yeast, resulting in substantial production of dihydroartemisinic acid in fermenters (Immethun et al., 2013; Paddon et al., 2013; Tang et al., 2014; Table 4). This dihydroartemisinic acid can subsequently photo-chemically be converted to (dihydro)artemisinin (Paddon and Keasling, 2014). Some of the issues that play a role in the potential boosting of artemisinin production in *A. annua* as discussed above, also play a role in boosting artemisinin production in heterologous hosts. For instance, high activity of *HMGR* is also important for the ectopic production of artemisinin in yeast (Pitera et al., 2007; Ye and Bhatia, 2012; Tang et al., 2014) (Table 4).

**TABLE 4:** *De novo* production of ART precursor via synthetic biology.

No.	Host	Gene(s)	Yield	References
1	*Saccharomyces cerevisiae*	Amorpha-4,11-diene synthase	Plasmid and genome-transformed produced 0.6 and 0.1 mg/l amorphadiene	Lindahl et al. (2006)
2	*Saccharomyces cerevisiae*	Mevalonate pathway, amorphadiene synthase, cytochrome P450 monooxygenase	≥ 100 mg/l artemisinic acid	Ro et al. (2006)
3	*Saccharomyces cerevisiae*	Amorphadiene synthase, amorphadiene oxidase, and cytochrome P450 reductase	250 mg/l (in shake-flask) and 1000 mg/l (in bioreactors) artemisinic acid	Ro et al. (2008)
4	*Saccharomyces cerevisiae*	Mevalonate pathway, overexpression of related genes	> 40000 mg/l amorphadiene	Westfall et al. (2012)
5	*Saccharomyces cerevisiae*	Complete biosynthetic pathway	25000 mg/l artemisinic acid	Paddon et al. (2013)
6	*Escherichia coli*	Expression of a synthetic amorpha-4,11-diene synthase and the mevalonate isoprenoid pathway from *Saccharomyces cerevisiae*	24 mg caryophyllene equivalent/l amorphadiene	Martin et al. (2003)
7	*Escherichia coli*	Nine genes from mevalonate pathway	500 mg/l amorphadiene	Newman et al. (2006)
8	*Escherichia coli*	Overexpression of mevalonate pathway genes	> 25000 mg/l amorphadiene	Tsuruta et al. (2009)
9	*Escherichia coli*	Amorphadiene biosynthetic pathway genes	293 mg/l/OD_600_ at 75h amorphadiene	Anthony et al. (2009)
10	*Escherichia coli*	Engineered substrate promiscuous P450_BM3_	250 mg/l amorphadiene	Dietrich et al. (2009)
11	*Escherichia coli*	Mevalonate pathway genes	235 mg/l amorphadiene	Wu T. et al. (2011)

*All units are converted to milligram per liter (mg/l).*

## Conclusion

Whether artemisinin can be used to not only combat malaria, but also other diseases, including those acting on a pandemic scale, will very much depend on further validation of the efficacy of artemisinin in these other diseases and on how this compound can cost-effectively be made available to the community. Multiple and complementary approaches may be necessary to boost synthesis capacity, varying from the transformation of *A. annua* itself to boost artemisinin yield, to investment into heterologous production platforms that may be easier to scale up. At the same time, we should not ignore the lessons learned from monotherapy in combating disease, as this may result in the emergence of artemisinin-resistance, as currently happening for malaria. Therefore, both for malaria, as for the potential of artemisinin in combating COVID-19 and other viral infections ACTs or triple artemisinin-based combination therapies (TACTs) may be required to prevent the rise of artemisinin resistant disease variants.

## Author Contributions

ABK conceptualized the review. KF-K, AR, AB, ARK, and ABK wrote the manuscript. ABK, ARK, and AB reviewed the manuscript. KF-K, AR, and ABK did the figures visualization. All authors contributed to the article and approved the submitted version.

## Conflict of Interest

The authors declare that the research was conducted in the absence of any commercial or financial relationships that could be construed as a potential conflict of interest.

## Publisher’s Note

All claims expressed in this article are solely those of the authors and do not necessarily represent those of their affiliated organizations, or those of the publisher, the editors and the reviewers. Any product that may be evaluated in this article, or claim that may be made by its manufacturer, is not guaranteed or endorsed by the publisher.

## Footnotes

1
https://clinicaltrials.gov/

2
https://www.chictr.org.cn/
